# Cellular Antioxidant Properties of *Ischnoderma Resinosum* Polysaccharide

**DOI:** 10.3390/molecules27227717

**Published:** 2022-11-09

**Authors:** Caiyu Liao, Liyan Wu, Wenting Zhong, Qinhua Zheng, Weijian Tan, Kexin Feng, Xiaolin Feng, Fanxin Meng

**Affiliations:** 1 College of Pharmacy and Food Science, Zhuhai College of Science and Technology, Zhuhai 519041, China; 2 College of Life Science, Jilin University, Changchun 130012, China

**Keywords:** polysaccharides, *Ischnoderma resinosum*, erythrocyte, HepG2 cells, oxidative damage, antioxidant

## Abstract

A predominant polysaccharide isolated from *Ischnoderma resinosum* underwent evaluation for its capacity to scavenge free radicals and its potential antioxidant properties at a cellular-oriented level. This proved that *Ischnoderma resinosum* polysaccharide (IRP) remarkably curtailed AAPH-induced erythrocyte hemolysis through the inhibition of the generation of ROS (*p* < 0.05). Rather, it caused the restoration of intracellular antioxidant enzyme (SOD, GSH-Px, and CAT) activities at an acceptable pace and the silencing of intracellular MDA formation, as well as the rescaling of LDH leakage. Furthermore, a model of oxidative stress in HepG2 cells was established by adopting 400 μM of hydrogen peroxide, which suggested that IRP manifests promising antioxidant activity. Notably, after the intervention of IRP in the H_2_O_2_-induced HepG2 cells, there was a statistical elevation in cell survivability (*p* < 0.05). IRP diminished the morphological alterations in the nucleus and decreased the secretion of ROS (*p* < 0.05), with a dose-dependent abrogation of apoptosis (*p* < 0.05). Consequently, IRP, which was isolated and purified, was able to scavenge free radicals and possessed favorable antioxidant activity that could dampen the occurrence of oxidative stimulation and effectively alleviate the AAPH-induced erythrocyte hemolysis and H_2_O_2_-induced oxidative damage in HepG2 cells. This provides a basis and theoretical reference for the development and utilization of IRP as a natural antioxidant, with emphasis on the exploitation of environmentally friendly and cost-effective antioxidants.

## 1. Introduction

People have discovered a variety of extremely physiologically active macrofungi, which are enormous, high-value-added fungi that include a great number of unique chemical structural components [1]. There is a huge variety of *polypores* in edible fungi that have medicinal value. More than 60 different types of *polypores*, including *Wolfiporia cocos*, *Polyporus umbellatus*, *Polyporus mylittae*, *Trametes Versicolor*, and others, are currently known to have medicinal use. This type of polypore is distinguished by a wealth of natural resources and ease of artificial cultivation, making it simple to create and use [2]. *Ischnoderma resinosum* (IR), a ligninolytic fungus, belongs to the order *Polyporales* and the family *Ischnoderma*, with large fruiting bodies distributed all over the world [3]. M A Fitra found that, as an edible fungus, the nutrient content of the fruiting body of the rosin wrinkled skin fungus was low in protein and fat content, and the proportion of carbohydrates was 3.10% [4].

Reactive oxygen species (ROS) function as signaling molecules in cells but are also believed to be an unavoidable and toxic byproduct of aerobic metabolism [5]. The harmful effects of excess ROS or oxidative stress have been reported to eventually lead to cell death [6]. Bioactive polysaccharides, which are the primary active ingredient in the majority of natural product extracts, can be a valuable natural source of drugs. Under oxidative stress conditions, some bioactive polysaccharides reduce the levels of ROS and the related peroxidation products in cells and animal models. Further to this, bioactive polysaccharides protect against oxidative damage by increasing the activity of various antioxidant enzymes, modulating gene expression and regulation, and controlling stress-related signaling events [7]. As a result, fungal polysaccharides are becoming more popular as free radical scavengers. Since oxidative stress is a dynamic balance between free radical production and scavenging, chemical methods evaluated in vitro in cellular models using the body’s cells as carriers have a more comprehensive network of active effects, and there is now some acceptance for both. These methods include DPPH radical, superoxide anion, hydroxyl radical, and ABTS radical scavenging [8,9,10]. As a result, models of cellular oxidative stress and free radical scavenging are both useful for examining the antioxidant activity of polysaccharides and their mode of action. Without a nucleus, erythrocytes can withstand oxidative stress and preserve osmotic equilibrium thanks to a functioning enzymatic nd nonenzymatic metabolic system [11]. Meanwhile, the H_2_O_2_-induced oxidative stress model in HepG2 cells has been widely accepted [12].

At present, there are only a few studies on *Ischnoderma resinosum* polysaccharide (IRP) and its antioxidant activity. In this study, the in vitro antioxidant activity of IRP was examined using a combination of chemical techniques (DPPH, ABTS, hydroxyl, and superoxide anion radical scavenging assay) as well as the erythrocyte hemolysis model caused by AAPH and the oxidative stress model caused by H_2_O_2_ in HepG2 cells. In order to clarify the putative antioxidant mechanism of IRPs at the cellular level, changes in ROS, MDA, LDH, and antioxidant enzymes (such as CAT, SOD, and GSH-Px) in erythrocytes and ROS, nuclei, and apoptosis in HepG2 cells following IRP therapy were also examined.

## 2. Results

### 2.1. FT-IR Analysis of IRP

**Figure 1:**
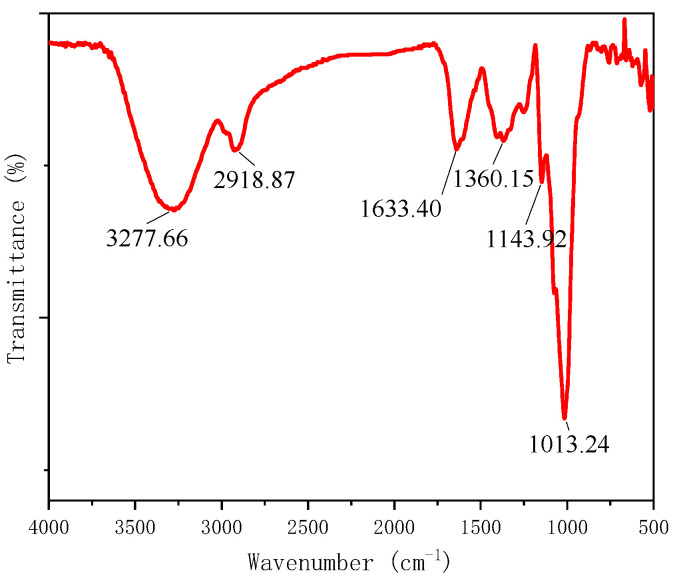
The infrared spectrum of IRP (scan interval: 500~4000 cm^−1^).

Fourier transform infrared spectroscopy (FT-IR) is one of the most useful techniques for identifying the structure of polysaccharides and is based on the analysis of characteristic wave numbers to determine structural information, such as polysaccharide functional groups and sugar ring conformations [13]. As seen in Figure 1, IRP appears as a strong and broad characteristic absorption peak in the 3200~3500 cm^−1^ range and a faint absorption peak near 2900 cm^−1^. These absorption peaks are attributed to the stretching vibrations of −OH and CH, respectively, with CH also showing a weaker absorption peak around the 1400 cm^−1^ range due to deformation vibrations. The strong and broad absorption peaks around 1030 cm^−1^ are the stretching vibrations between the C−O and C−C bonds and the bending vibrations of C−OH. In conclusion, the IRP absorption peak at 1633 cm^−1^, which is attributed to the carboxylate ion (COO−) stretching vibration, also confirms the presence of glyoxalate in the IRP. The IR spectroscopy results point to IRP having the functional group of a polysaccharide and being a typical polysaccharide [14].

### 2.2. UV Spectroscopic Analysis

**Figure 2:**
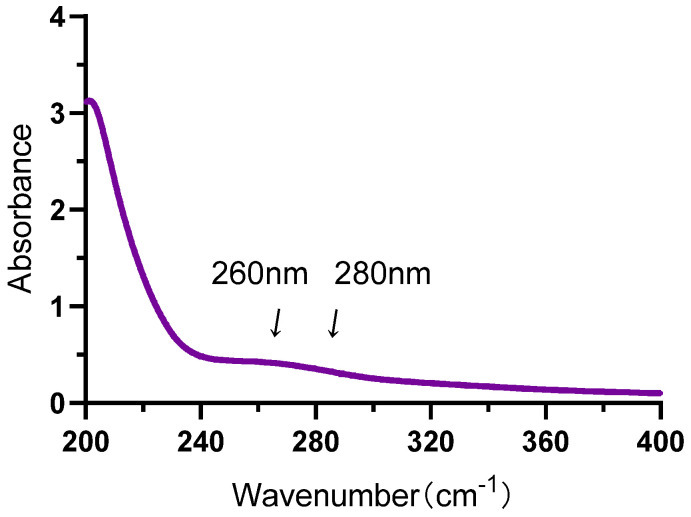
UV full-band scan of IRP; infrared spectrum of IRP (scan interval: 200~400 cm^−1^).

In this work, the UV spectrum of the IRP was scanned at full wavelength, and the results are shown in Figure 2. IRP has no significant absorption peaks at 260 nm and 280 nm, indicating that the sample contains no or minor protein and nucleic acid-like impurities [15]. 

### 2.3. Congo Red Analysis

**Figure 3:**
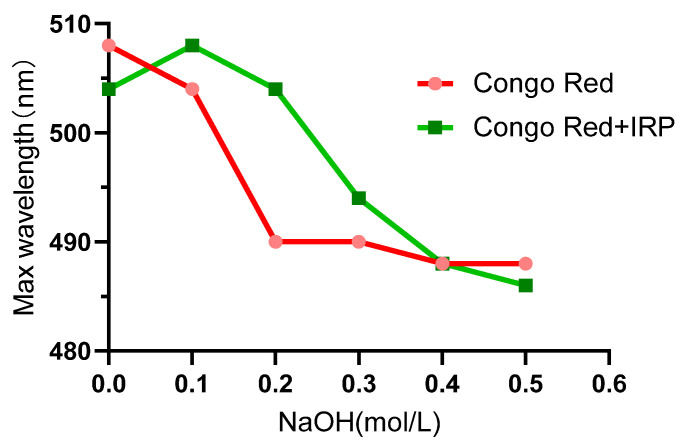
Maximum absorption wavelength of IRP complexed with Congo red at different concentrations of NaOH.

The Congo red dye can form complexes with polysaccharides containing triple helix structures as well as shift the maximum red absorption wavelength (λ_max_) in the full wavelength scan of UV compared to Congo red alone. Accompanied by an increase in the concentration of NaOH in the mixed solution, the structure of the complex was disrupted under alkaline conditions, where the complex was unlocalized from a triple helix to a single helix and then from a single helix to a random curl, eventually leading to the disruption of the triple helix structure. In this procedure, the λ_max_ of the complex increases and then decreases dramatically so that the triple helix structure of the polysaccharide can be verified by the Congo red test. In this case, the Congo red method was used to elicit the conformational changes of the IRP-Congo red complex when the NaOH in the mixed solution reached 0~0.5 M. At a NaOH concentration of 0.1 M, the IRP-Congo red complex appeared redshifted compared to the Congo red λ_max_. When the NaOH concentration was increased from 0.1 M to 0.3 M, the λ_max_ of the IRP-Congo red complex decreased promptly (Figure 3), implying that the IRP contains a triple helix structure. Typically, the carboxyl or triple helix structures not only confer enhanced biological activity to the polysaccharide, but polysaccharides predicated on the triple helix structure may possess unique functional properties that are unmatched by polysaccharides in general; therefore, IRPs may have good biological activity and some specific functions [16].

### 2.4. Monosaccharide Composition Analysis

**Figure 4:**
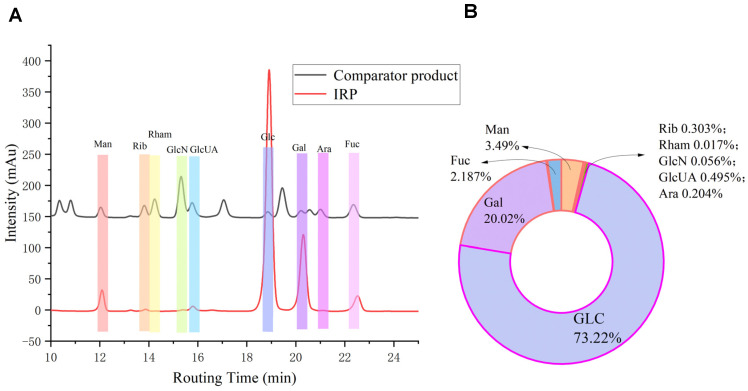
HPLC chromatogram of monosaccharide standards and IRP (**A**), characteristic peaks: 1. gully aldehyde; 2. mannuronic acid; 3. mannose; 4. ribose; 5. rhamnose; 6. glucosamine; 7. glucuronic acid; 8. galacturonic acid; 9. glucose; 10. aminogalactose; 11. galactose; 12. xylose; 13. arabinose; and 14. l-fucose. (**B**) Molar percentage pie chart of IRP monosaccharide composition.

IRP is composed of mannose, ribose, rhamnose, glucosamine, glucuronic acid, glucose, galactose, arabinose, and L-fucose, as shown in Figure 4, and was obtained by the retention time of each single-labeled peak, as well as the chromatographic peak area. The molar percentages are 3.496: 0.303: 0.017: 0.056: 0.495: 73.224: 20.018: 0.204: and 2.187. The sum of mannose, glucose, and galactose is more than 96% of the total. It is mainly related to the type of monosaccharide and its ratio, such as glucose and galactose, which are necessary for the antioxidant activity of polysaccharides. Our preliminary results indicate that glucose and galactose are the major fractions in IRP, along with small amounts of mannose and fucose. Fungal polysaccharides are chiefly composed of mannose, glucose, and galactose, and IR is a species of common edible fungus. The graphical structure of the above monosaccharides indicates that IRP contains mainly mannose, glucose, galactose, and a small amount of rock sugar, which is in agreement with the literature [17].

### 2.5. Determination of the Ability of IRP to Scavenge ABTS, Hydroxyl, DPPH, and Superoxide Anion Radicals 

**Figure 5:**
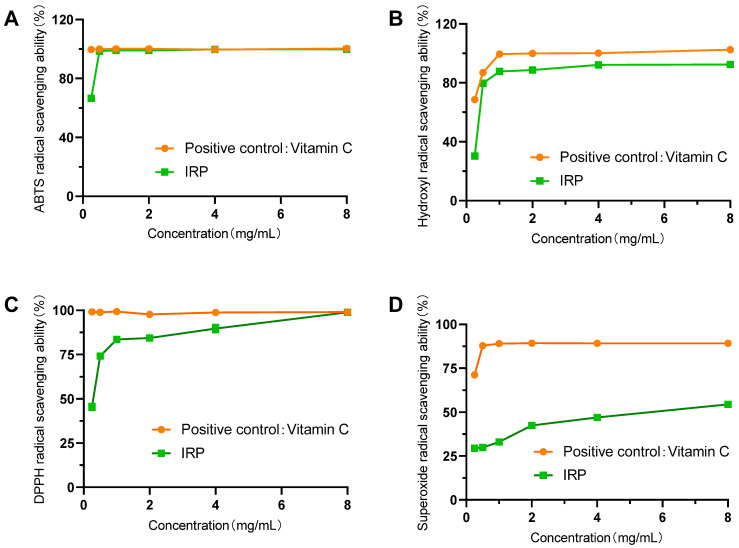
The radical scavenging ability of IRP (**A**): ABTS radical scavenging ability of IRP; (**B**): Hydroxyl radical scavenging ability of IRP; (**C**): DPPH radical scavenging ability of IRP; (**D**): Superoxide radical scavenging ability of IRP).

In Figure 5A, the scavenging rate of ABTS radicals by IRP increased with an increase in sample mass concentration in a concentration-dependent manner. The scavenging rate was 98.63% when IRP was 0.5 mg/mL. This was not different from the positive control, V_C_. From the results, it is clear that IRP has a strong ability to scavenge ABTS free radicals. 

In this assay, the scavenging activity of different concentrations of IRP on the hydroxyl radicals was measured. It is shown in Figure 5B that the scavenging rate of the hydroxyl radicals by IRP increased with an increase in sample quality concentration, and the scavenging effect of IRP increased slowly with an increase in concentration above 1 mg/L and reached the maximum at 4 mg/mL, with a maximum scavenging rate of 92.10%. The scavenging capacity of the positive contrast, V_C_, was 100% at this time. The results clearly show that IRP has a strong ability to scavenge hydroxyl radicals.

As seen in Figure 5C, the DPPH scavenging activity of IRP was noticeable at all concentrations tested and was positively correlated with concentration. The increase in the DPPH radical scavenging rate from 45.48% to 83.55% was greater when the sample mass concentration was increased from 0.25 mg/mL to 1 mg/mL. Subsequently, the scavenging rate of the DPPH radicals increased slowly with increasing sample mass concentration, reaching 98.86% at a sample mass concentration of 8 mg/mL. It was demonstrated that IRP has a well-defined ability to scavenge DPPH radicals. 

The scavenging of superoxide anions by IRP is shown in Figure 5D. The data show that the scavenging ability of IRP on superoxide anions was positively correlated with concentration. At lower concentrations (0.25–2 mg/mL), the IRP scavenging activity increased rapidly, while at higher concentrations (2–8 mg/mL), the IRP scavenging activity reached a maximum of 54.42%. IRP is less effective in scavenging superoxide anions than DPPH radicals and hydroxyl radicals.

### 2.6. Evaluation of Antioxidant Activity Using the AAPH-Induced Erythrocyte Model

#### 2.6.1. The Inhibitory Effect of IRP on AAPH-Induced Erythrocyte Hemolysis

**Figure 6:**
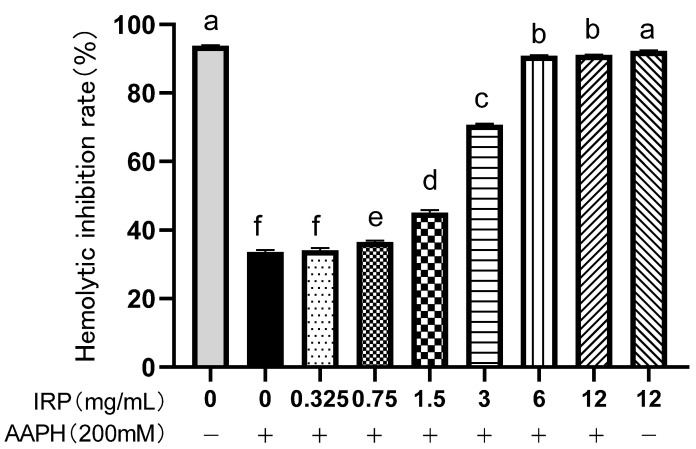
Inhibition of AAPH-induced erythrocyte hemolysis by IRP (the same group of lowercase letters, if containing an identical letter, is not a significant difference, where no identical letter is a significant difference (*p* < 0.05)).

As in Figure 6, the hemolytic inhibition of erythrocytes by AAPH was only 33.67%, which increased significantly to 90.91% as the IRP concentration increased from 0 to 6 mg/mL. When the concentration reached 12 mg/mL, the rate of hemolysis inhibition in the IPP was almost the same as in the control group (normal red cell group). This outcome reveals that IRP does not cause erythrocyte damage and has a beneficial capability to inhibit the AAPH-induced oxidative hemolysis of erythrocytes. On the other hand, in the absence of AAPH, the hemolysis inhibition rate of the erythrocytes treated with the highest concentration of IRP was similar to that of normal erythrocytes treated with PBS alone, implying that IRP is nontoxic and does not cause erythrocyte damage.

#### 2.6.2. Effects of IRP on AAPH-Induced Changes in ROS Level of Erythrocytes

**Figure 7:**
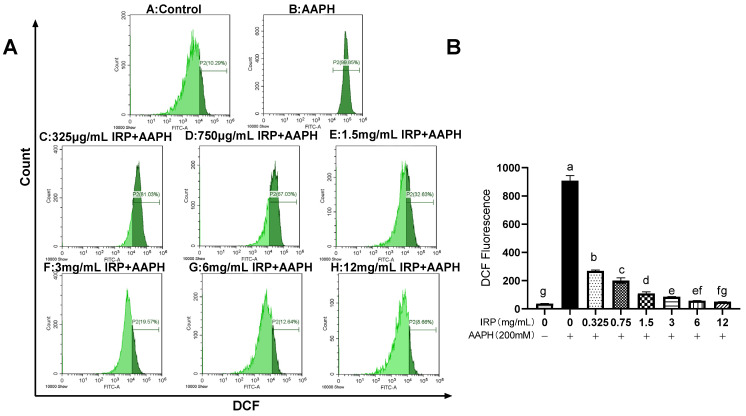
Effect of IRP on ROS levels after AAPH treatment of erythrocytes. (**A**): effect of IRP on intracellular reactive oxygen species (ROS) levels. (**B**): relative DCF fluorescence intensity. The same group of lowercase letters, if containing an identical letter, is not a significant difference, where no identical letter is a significant difference (*p* < 0.05).

The relative levels of intracellular ROS were analyzed by measuring the fluorescence intensity by flow cytometry. As in Figure 7, the fluorescence intensity of the ROS was comparable in 12 mg/mL IRP-treated erythrocytes when compared to the control, with the remaining groups having increased proportions, especially the 200 mM AAPH model group, which significantly (*p* < 0.05) promoted intracellular ROS production with a fluorescence intensity of up to 892.58 ± 29.45. When pretreated with different concentrations of IRP (0.325, 0.75, 1.5, 3, 6, and 12 mg/mL), the fluorescence intensity was reduced, and all of them significantly inhibited the production of ROS by cells during the action of AAPH, showing a dose-dependent relationship (*p* < 0.05). It is worth noting that the ROS fluorescence intensity level could be reduced by 73% at 0.325 mg/mL of IRP when compared to the model group. This trial confirmed that IRP could scavenge and inhibit intracellular ROS, with 12 mg/mL of IRP having the strongest ability to scavenge ROS and a 93% decrease in fluorescence intensity compared to the model group.

In this study, cell viability was significantly reduced, and intracellular ROS levels were significantly increased in the model group, indicating that the cells were in a state of oxidative stress after AAPH injury. It is speculated that IRP may inhibit the production of intracellular ROS to maintain oxidative homeostasis and ultimately restore cell viability.

#### 2.6.3. Influence of IRP on AAPH-Induced Antioxidant Enzyme Activity in Erythrocytes

**Figure 8:**
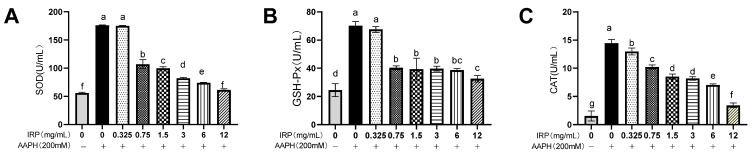
Effect of IRP on AAPH-induced antioxidant enzymes (SOD (**A**), GSH-Px (**B**), and CAT levels (**C**)) in erythrocytes (the same group of lowercase letters, if containing an identical letter, is not a significant difference, where no identical letter is a significant difference (*p* < 0.05)).

A 215% increase in cellular SOD (Figure 8A) activity following AAPH injury was observed when compared to the control group. SOD in those erythrocytes treated with 12 mg/mL IRP decreased by 186% compared to the model group with AAPH injury. The intracellular enzymatic activities of SOD, GSH-Px (Figure 8B), and CAT (Figure 8C) were enhanced considerably in erythrocytes upon their exposure to AAPH treatment, indicating a positive response of the cellular defense system to oxidative stress. Correspondingly, SOD, GSH-Px, and CAT activity was inhibited in those experimental groups in which the erythrocytes were pretreated with IRP as well as with AAPH in a dose-dependent manner, especially at high concentrations (12 mg/mL). Besides, the SOD, GSH-Px, and CAT activities in the subject group treated with IRP only were similar to those in the control group (normal erythrocytes). This implies that IRP reduces the AAPH-induced oxidative stress in erythrocytes, thereby allowing the activity of the antioxidant enzymes in erythrocytes to reach normal levels.

#### 2.6.4. Effects of IRP on AAPH-Induced Changes in LDH and MDA Level of Erythrocytes 

**Figure 9:**
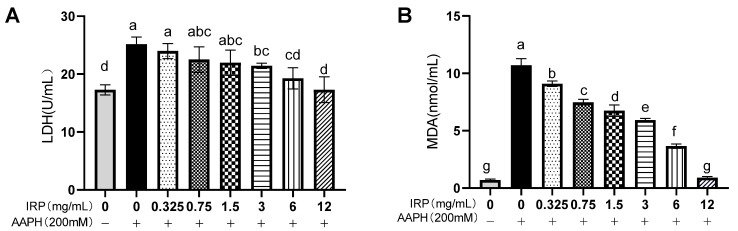
Effect of IRP on LDH (**A**) and MDA (**B**) in AAPH-induced erythrocytes (the same group of lowercase letters, if containing an identical letter, is not a significant difference, where no identical letter is a significant difference (*p* < 0.05)).

The LDH (Figure 9A) level was elevated by 31.28% in the model group compared to the control group (*p* < 0.05), while the prophylactic effect of 12 mg/mL of IRP reduced the LDH level by 31.19%, which was not measurable compared to the control group, indicating that the LDH largely returned to normal levels. Figure 9B illustrates the effect of IRP on MDA production in erythrocytes. Intracellular MDA accumulation was evident after the inhibition of the erythrocytes by AAPH in isolation. The MDA level increased from 0.71 ± 0.09 nmol/mg to 10.71 ± 0.57 nmol/mg in the control cell group and did not vary appreciably for several hours after exposure. This indicates that AAPH-treated erythrocytes underwent severe lipid peroxidation. In the dosed group protected with IRP, the MDA levels significantly (statistically) declined, with an explicit dose-dependent relationship. In all cases, the levels of MDA were not similar to those of the control erythrocytes treated with 12 mg/mL of IRP. 

### 2.7. Evaluation of Antioxidant Activity Using a Model of H_2_O_2_-Induced Oxidative Damage in HepG2 Cells

#### 2.7.1. Effect of Different Concentrations of IRP on HepG2 Cells

**Figure 10:**
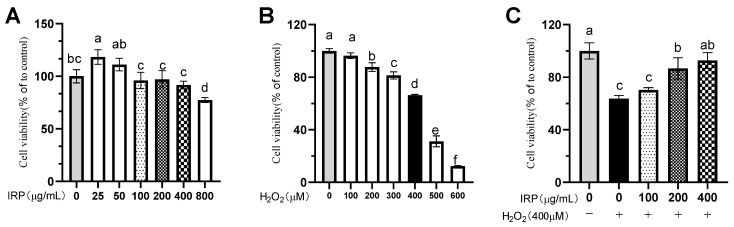
Effects of IRP on H_2_O_2_-induced cell viability. (**A**) Effect of different concentrations of IRP on HepG2 cells; (**B**) effect of different concentrations of H_2_O_2_ on HepG2 cells; **and** (**C**) preventive protection of IRP against H_2_O_2_-induced oxidative damage in a model of HepG2 cells. The same group of lowercase letters, if containing an identical letter, is not a significant difference, where no identical letter is a significant difference (*p* < 0.05).

Figure 10A plots the efficacy of different concentrations of IRP on the viability of HepG2 cells, from which it can be seen that, at low concentrations (25 and 50 μg/mL), IRP intervention in HepG2 cells for 24 h produces an effect. The IRP intervention in HepG2 cells at high concentrations (800 μg/mL) for 24 h produced toxic effects. That is why the 100, 200, and 400 μg/mL concentrations of IRP were chosen for the follow-up experiments to exclude the false positive and false negative effects of IRP on the HepG2 cell; this indicates that the coculture of IRP with HepG2 cells at the above concentration for 24 h has no impact on the HepG2 cells, and, therefore, the intervention concentration can be screened with this concentration gradient.

#### 2.7.2. Effect of Different Concentrations of H_2_O_2_ on HepG2 Cells

HepG2 cells were subjected to final concentrations of 100, 200, 300, 400, 500, and 600 μM of H_2_O_2_ medium for 4 h, and the changes in cell viability were studied. As seen in Figure 10B, after 4 h of exposure to different concentrations of H_2_O_2_, the viability of the HepG2 cells was severely impaired in all groups except for the 100 μM group (*p* < 0.05). Simultaneously, with an increase in H_2_O_2_ concentration, the cell viability gradually decreased, ensuring a sufficient number of cells in the later stage of the experiment. A H_2_O_2_ concentration of 400μM was selected for HepG2 cell damage in this experiment, at which time the cell viability was (66.21 ± 0.71) %.

#### 2.7.3. Protective Effect of IRP on H_2_O_2_-Induced Oxidative Damage in a Model of HepG2 Cells

Free radical production occurs in cells in the presence of oxidative damage triggers, yet some of this damage can potentially be repaired in the presence of suitable antioxidants. The effectiveness of IRP on the survival rate of the HepG2 cells with H_2_O_2_-induced oxidative damage is depicted in Figure 10C. The findings demonstrate that 100, 200, and 400 μg/mL IRP has a protective effect against H_2_O_2_-induced oxidative damage. Cell survival measurably increased for IRP at 200–400 μg/mL. It can be said that IRP can directly reduce the oxidative damage induced by H_2_O_2_ in HepG2 cells in the concentration range of 200~400 μg/mL and has an obvious protective effect on oxidative damage in HepG2 cells. The most potent protective effect was achieved by IRP at 400 μg/mL, reaching 92.73 ± 5.94%. Compared to the model group, IRP increased cell survival by 11%, 36%, and 46% at concentrations of 100, 200, and 400 μg/mL, respectively.

#### 2.7.4. Effect of IRP on ROS in a Model of H_2_O_2_-Induced Oxidative Damage in HepG2 Cells

**Figure 11:**
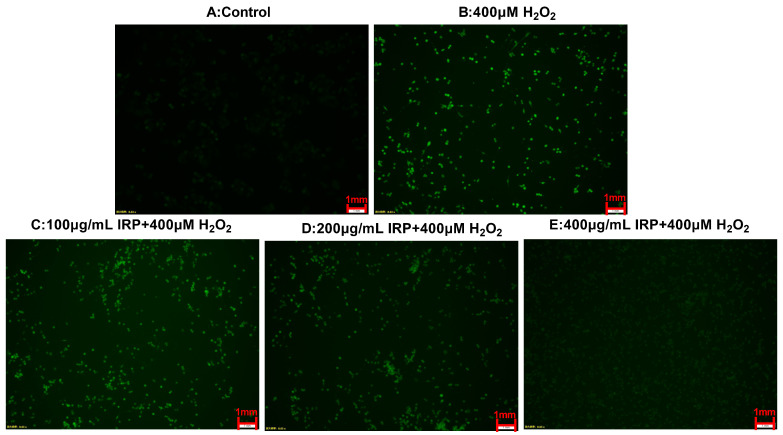
Effects of IRP on the H_2_O_2_-induced intracellular redox status.

**Figure 12:**
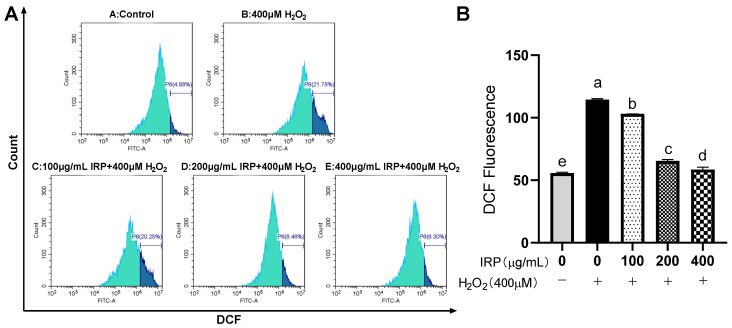
Effect of IRP on ROS levels in a model of H_2_O_2_-induced oxidative damage in HepG2 cells. (**A**) Effect of IRP on intracellular reactive oxygen species (ROS) levels. (**B**) Relative DCF fluorescence intensity. The same group of lowercase letters, if containing an identical letter, is not a significant difference, where no identical letter is a significant difference (*p* < 0.05).

The DCFH-DA method was used to detect intracellular ROS. The results of this experiment are shown in Figure 11 and Figure 12. Compared to the control group, the fluorescence intensity of the other group was markedly enhanced, and the amount of ROS visibly increased, a demonstration that H_2_O_2_ can induce a higher production of ROS in HepG2 cells [18,19]. Compared with the control group, the fluorescence intensity reached 114.44 ± 0.77, and the fluorescence intensity of the experimental group (100, 200, 400 μg/mL) gradually reduced, indicating that the early addition of IRP could effectively inhibit the production of ROS by 9.97, 42.83, and 48.80%, respectively. The control group also had some ROS content; 400 μg/mL of IRP restored almost the same fluorescence intensity as the control group, thus protecting the cells from ROS-induced damage. As can be seen from the graph, the control group has a high cell density, clear cytoplasm, and the weakest fluorescence intensity, indicating a low level of ROS. After H_2_O_2_ stimulation, cell density decreased significantly, intracellular fluorescence intensity became greater, and the ROS levels notably increased. The addition of IRP intervention increased cell density and reduced fluorescence intensity. IRP reduced intracellular ROS levels, possibly because IRP inhibits ROS production or scavenges ROS. 

#### 2.7.5. Effect of IRP on Cell Nucleus in H_2_O_2_-Induced Oxidative Damage Model of HepG2 Cells by DAPI Staining

**Figure 13:**
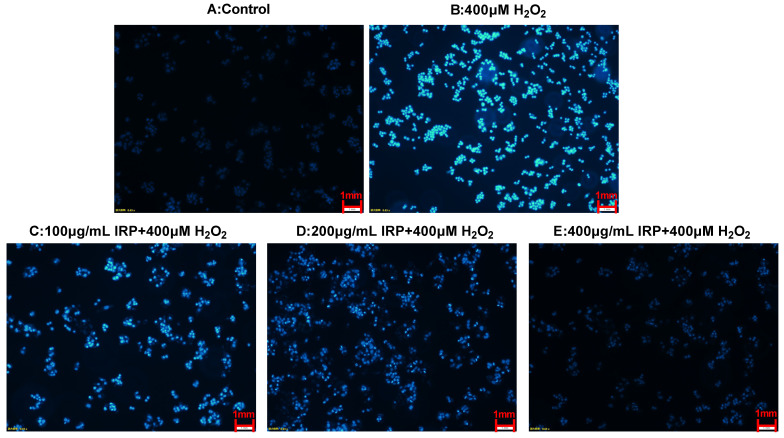
DAPI staining to observe the effect of IRP on the nucleus in a model of H_2_O_2_-induced oxidative damage in HepG2 cells.

Morphological changes in the nuclei were observed using DAPI staining. As can be seen from Figure 13, the cells in the control group showed a very weak blue fluorescence, proving that the nuclear structure of the cells was normal. Upon induction by H_2_O_2_, the proportion of live HepG2 cells reduced and the chromatin nuclei condensed, including the appearance of the membrane vesicles and granular apoptotic vesicles, which is characteristic of apoptosis [20]. In contrast, after IRP treatment, the fluorescence intensity diminished gradually, and the number of apoptotic cells was reduced in a dose-dependent manner. From the above results, it is clear that IRP can inhibit H_2_O_2_-induced apoptosis.

#### 2.7.6. The Effect of IRP on Apoptosis in H_2_O_2_-Induced Oxidative Damage Model of HepG2 Cells

**Figure 14:**
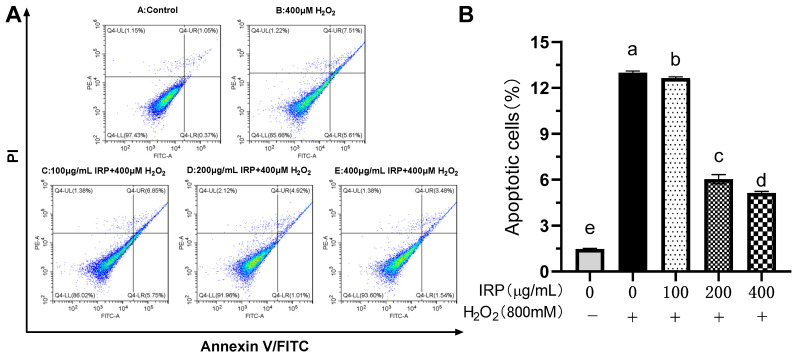
Effect of IRP on apoptosis in a model of H_2_O_2_-induced oxidative damage in HepG2 cells. (**A**) Flow scatter plot of apoptosis detected by flow cytometry; (**B**) Statistical plot of apoptosis detected by flow cytometry (the same group of lowercase letters, if containing an identical letter, is not a significant difference, where no identical letter is a significant difference (*p* < 0.05)).

Figure 14A plots the scatter distribution of apoptosis for H_2_O_2_-induced oxidative damage in HepG2 cells by IRP. A total of 10,000 cells were tested, with the number of normal cells in the lower left quadrant, LL, and the number of early apoptotic cells in the lower right quadrant, LR. UR, in the upper right quadrant, is the number of cells with late apoptosis; UL, in the upper left quadrant, is the number of cells with necrotic cells, and the apoptosis rate is the total apoptosis rate for both early and late stages. As can be seen from the graph, treatment with IRP resulted in a significant reduction in the number of both early and late apoptotic cells in the HepG2 cells compared to the model group. The specific apoptotic rate statistics are shown in Figure 14B, where the cells were damaged by H_2_O_2,_ and the apoptosis rate increased by 11.70%. On the contrary, IRP, at 100, 200, and 400 μg/mL, reduced the apoptosis rate by 2.719, 53.515, and 60.467%, respectively, and showed a dose-dependent relationship, which was statistically significant when compared with the model group. These figures reflect a major increase in both early and late apoptosis and the appearance of necrotic cells after 4 h of H_2_O_2_ damage, while the preventive effect of the IRP reduced the early and late apoptosis rates. Thus, IRP inhibits H_2_O_2_-induced apoptosis. Among the present studies, IRP not only evinced in vitro antioxidant activity but also significantly increased cell viability in the H_2_O_2_-induced HepG2 cells; it also reduced cellular oxidative stress and maintained cellular redox homeostasis by inhibiting the production and activity of ROS.

## 3. Materials and Methods

### 3.1. Materials

*Ischnoderma resinosum* grows on elm trees, sessile, or spreading to revolute, and is subcorky, with a semi-circular cap and a rusty to dark brown colored surface, with radial wrinkles and a thin epidermal layer. The flesh is wood-colored, and the fruiting bodies are harvested after they dry. Harvested in September 2021 in the Jilin Province, China (Production lot: Tianshui Zhongxing Edible Mushroom Technology Co., Ltd., Jilin, China). They were identified by gene sequencing by Bioengineering Co. (Shanghai, China) as *Ischnoderma resinosum.* Dried at 50 °C, crushed, and sieved through 100 mesh to produce IR dry powder.

HepG2 was purchased from the Typical Culture Collection Committee Cell Bank, Chinese Academy of Sciences, Shanghai, China; 2,2’-Azo(di isobutyl amidine dihydrochloride). AAPH was purchased from Source Leaf Ltd., Shanghai, China; DMEM, PBS purchased from Hyclone, Logan, CA, USA; Fetal bovine serum (FBS) was purchased from Pronoxa, Wuhan, China; DPPH radical, superoxide anion, ABTS radical, OH radical, catalase (CAT), superoxide dismutase (SOD), lactate dehydrogenase (LDH), and malondialdehyde (MDA) kits were purchased from Nanjing Jiancheng Biotechnology Co, Nanjing, China; 4% Tissue Cell Fixative, DAPI, Apoptosis (Annexin V/PI), ROS Kit, Solebro Technologies Ltd., Beijing, China; Cell Counting Kit-8 (CCK8) was purchased from Beyotime Biotechnology Ltd., Shanghai, China; hydrogen peroxide was purchased from Sinopharm Chemical Reagent Co, Shanghai, China.

### 3.2. Extraction and Purification of IRP

IRP was extracted by ultrasonic microwave coextraction [21] at 200 W ultrasonic power, 200 W microwave power, and 1:50 material-to-liquid ratio for 40 min. After deproteinization and overnight alcohol precipitation, crude polysaccharide was obtained, concentrated, and dried to 5.0 mg/mL crude extract and filtered through a 0.45 μm microporous membrane. Purified by DEAE-Sepharose Fast Flow column chromatography and Sephadex G-150 dextran gel column with dynamic adsorption; the filtrate was eluted with ultrapure water, collected again, concentrated by rotary evaporation at 55 °C, dialyzed, and dried at 3500 Da to obtain IRP. The content of the polysaccharides was determined by the phenol-sulfuric acid method using D-glucose as the standard reference [22]. The purity (%) was calculated using the following equation, according to previous studies [23,24,25]. The experimental results demonstrated a purity of (85.40 ± 1.58) % for IRP.

**Figure 15:**
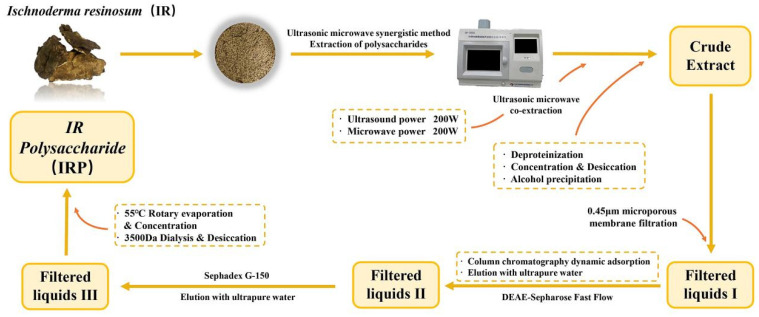
The procedure for the extraction and purification of IRP.

Purity (%) = A_2_/A_1_ × 100

where A_1_ is the weight of dried crude polysaccharides (g), and A_2_ is the content of polysaccharides (g). Extraction, and purification procedures for IRP is illustrated in Figure 15.

### 3.3. Structural Analysis

#### 3.3.1. FT-IR Analysis of IRP

The IRP was mixed with a certain amount of potassium bromide powder and then pressed (2 mg of sample per 200 mg of potassium bromide). The pressed tablets were placed in a Fourier transform infrared (FTIR) spectrometer and scanned in the infrared range of 400~4000 cm^−1^ to obtain the IR absorption spectrum of IRP [13].

#### 3.3.2. Ultraviolet Spectroscopic Analysis

IRP was formulated as a 0.5 mg/mL solution and scanned between 200~400 nm for UV spectra and observed for absorption peaks between 260 nm and 280 nm [15].

#### 3.3.3. Congo Red Analysis

A Congo red method was used to determine and analyze whether IRP has a triple helix structure [14]. We weighed 5.0 mg of IRP, added 2 mL of distilled water, dissolved this completely, and then added 2 mL of Congo red reagent (80 μmol/L) and mixed thoroughly; NaOH solution was added so that the final concentration of NaOH was 0, 0.1, 0.2, 0.3, 0.4, and 0.5 M, respectively. The maximum absorption wavelength of the solution for each NaOH concentration was measured in a UV-Vis spectrophotometer, scanning between 200–800 nm. Without the polysaccharide samples, the maximum absorption wavelengths were measured under blank conditions in the same way.

#### 3.3.4. Monosaccharide Composition Analysis

The monosaccharide composition was measured using the PMP-HPLC method [14]. We dissolved IRP in 5 mL of 2 mol/L TFA (100 °C, 2 h), cooling to 26 °C, spiral dried the TFA under decompression, mixing with 1 mL of methanol, and continued to evaporate, repeating three times to completely discard the residual TFA; we solubilized the residue fully by adding 1 mL of 0.3 mol/L NaOH solution for the polysaccharide hydrolysis solution. The product was derivatized with PMP, blended, and reacted at 70 °C for 2 h to produce the final PMP derivative, which was cooled over the organic membrane and transferred into the injection vial for measurement. All monosaccharide standards and mixes were also derivatized, as mentioned above, and transferred into injection vials for detection using liquid chromatography. The operating conditions were as follows: amino column (4.6 × 250 mm, 5 μm) at 1.0 mL/min and an injection volume of 5 μL. Gully aldehyde, mannuronic acid, mannose, ribose, rhamnose, glucosamine, glucuronide, galacturonic acid, glucose, aminogalactose, galactose, xylose, arabinose, fucose were used as monosaccharide standards.

### 3.4. Determination of the Ability of IRP to Scavenge Hydroxyl, Superoxide Anion, ABTS, and DPPH Radicals 

Hydroxyl radicals are known to be one of the most intense free radicals and can lead to oxidative damage to adjacent biomolecules, leading to cytotoxicity, mutagenesis, carcinogenesis, and other diseases. Superoxide radicals are weak radicals and are a major contributor to oxidative stress damage. The DPPH radical scavenging assay is a useful tool for determining the antioxidant activity of polysaccharides [26,27]. In order to assess the antioxidant capacity of four different free radicals, DPPH, superoxide anion, hydroxyl radical scavenging, and ABTS, their radical scavenging activity was measured, referring to the kit instructions for experimental manipulation. Vitamin C (V_C_) was used as a positive control, and IRP concentrations were consistent with V_C_ concentrations (0.25, 0.5, 1, 2, 4, and 8 mg/mL).

### 3.5. Evaluation of Antioxidant Activity Using the AAPH-Treated Erythrocyte Model

#### 3.5.1. AAPH-Treated Erythrocyte Hemolysis Assay

Erythrocytes are extremely susceptible to oxidative stress, leading to hemolysis and the release of free hemoglobin into the plasma due to their high hemoglobin content and large cell membrane surface area. Based on the method from [28], with minor modifications, 10 mL of anticoagulated sheep blood was centrifuged at 600× *g* for 10 min at 4 °C to remove the serum and separate the erythrocytes. The erythrocytes were eluted three times with PBS (pH 7.4) to clarify the supernatant. Preparation of a 20% erythrocyte suspension was carried out by mixing erythrocytes with four times the volume of PBS. After dissolving IRP in PBS, 200 μL of erythrocyte suspension was mixed with 200 μL of IRP (0.325, 0.75, 1.5, 3, 6, and 12 mg/mL) in the dosing group and 200 μL of PBS was added to the control and model groups; after 30 min of incubation at 37 °C, 200 mM of AAPH 400 μL was added to the dosing and mod groups, and 400 μL of PBS was added to the control group, which were then mixed thoroughly and incubated for 2 h at 37 °C. When finished, all samples were diluted with 8 mL of PBS and centrifuged (600× *g*, 10 min, 4 °C). An amount of 200 μL of the supernatant was placed in a 96-well plate, and the absorbance (A) was measured at 540 nm. Complete hemolysis was obtained by adding 8 mL of ultrapure water to the cell suspension, and the absorbance was measured under the same conditions (B). The hemolysis rate is calculated by the following formula:

$$\mathrm{Hemolysis}\;\mathrm{inhibition}\;\mathrm{rate}\;(\%)=(1-\frac{A}{B})\times \;100\%$$

#### 3.5.2. Determination of ROS Generation

In this article, the fluorescein-labeled dye DCFH-DA was used to detect intracellular ROS levels. DCFH-DA itself is not fluorescent; however, it can penetrate cell membranes and be hydrolyzed to DCFH by intracellular esterases. The reduced form of DCFH is vulnerable to oxidation by ROS and to the highly fluorescent DCF [29]. The supernatant was discarded after three washes with PBS, and the erythrocytes were resuspended at five times the volume of PBS, after dilution. In addition, 100 μL was aspirated from the suspension to assay the intracellular ROS levels. After centrifugation (600× *g*, 10 min, 4 °C), 200 μL of 10 μM DCFH-DA was added and then incubated for 20 min at 37 °C in the dark. After completion of incubation, the cells were washed and resuspended in 600 μL PBS. Then, the intracellular ROS content was determined by measuring the fluorescence intensity of the cells using flow cytometry (Beckman, CA, USA).

#### 3.5.3. Analysis of Intracellular SOD, GSH-Px, CAT, LDH, and MDA

Erythrocytes were pretreated with IRP and then incubated with AAPH (200 mM) as described above. Afterward, the samples were centrifuged at 600× *g* for 10 min at 4 °C, and the supernatant was collected to determine the rate of LDH release. The erythrocytes were then rinsed twice with PBS; cold double ultrapure water was added to induce complete hemolysis of the erythrocytes, and MDA, SOD, GSH-Px, and CAT were quantified. All of these were measured according to the instructions of the assay kit, briefly described as follows.

The pyruvate assay is based on the reaction of pyruvate with 2,4-dinitrophenylhydrazine (DNPH) to form a red-brown dye; absorbance is measured at 450 nm using a Microplate Reader (Epoch, Bio Tek, Shoreline, WA, USA). The LDH activity in the assay is measured using the reaction system of the assay kit, in which lactic acid reacts with LDH to produce pyruvate, and then pyruvate is quantified.

The MDA content was estimated by the thiobarbituric acid method (TBA), based on the absorbance at 532 nm of the red-violet complex, which forms with MDA.

The SOD activity of the erythrocytes was measured in the O_2_^−^ production system. In this system, O_2_^−^ is produced by the reaction of xanthine with xanthine oxidase, which oxidizes hydroxylamine to produce nitrite, which, in turn, reacts with a dye-forming reagent added, according to the test kit, to produce a reddish-purple color. The SOD activity is calculated by measuring the absorbance at 450 nm to obtain the inhibition rate of O_2_^−^. 

The reaction of glutathione (GSH) with 5,5′-dithiobenzoic (2-nitrobenzoic acid) (DTNB) occurs to form a stable yellow substance, which can be quantified at 412 nm. Because GSH can be processed by GSH-Px to produce oxidized glutathione (GSSG), the decrease in absorbance corresponds to an increase in GSH-Px activity. Calculated according to the kit’s GSH-Px activity instructions.

CAT activity was determined using the ammonium molybdate method. The addition of ammonium molybdate suspends the breakdown of hydrogen peroxide by catalase, and the unbroken hydrogen peroxide reacts with ammonium molybdate to produce a yellowish product. The absorbance can be measured at 405 nm, and CAT viability can be calculated by measuring the shade of yellowish color.

### 3.6. Evaluation of Antioxidant Activity Using a Model of H_2_O_2_-Induced Oxidative Damage in HepG2 Cells

#### 3.6.1. Cell Cultures

HepG2 cells were cultured in 25 cm^2^ flasks in a medium containing 1% penicillin-streptomycin, 10% fetal bovine serum, and 89% DMEM in an incubator at 37 °C and 5% CO_2_. Cells were observed daily, and when the cells reached 80–90% fusion, they were digested with 0.25% trypsin for passaging culture.

#### 3.6.2. Effect of IRP on Cell Viability

Cells in satisfactory growth conditions and a logarithmic growth phase were inoculated in 96-well plates and cultured for 24 h or overnight until the cells were adherent. Cells were incubated with different concentrations of IRP medium (0, 50, 100, 200, 400, and 800 μg/mL) for 24 h. Cell viability was determined using the CCK-8 method [30,31].

#### 3.6.3. Screening of H_2_O_2_ Action Concentrations

H_2_O_2_ is an endogenous oxidant that can freely cross cell membranes and is commonly used in the establishment of oxidative stress injury models; the H_2_O_2_-induced oxidative injury model in HepG2 cells was established [12]. In order to test for H_2_O_2_-induced cytotoxicity in HepG2 cells, the cells were treated with increasing concentrations of H_2_O_2_ (0, 100, 200, 300, 400, 500, and 600 μM) for 4 h, selecting the concentration of H_2_O_2_ that would result in essentially half the cell viability. Cell viability was determined using the CCK-8 method.

#### 3.6.4. Preventive Protection of IRP against H_2_O_2_-Induced Oxidative Damage in HepG2 cells

Experimental groups: control group: complete medium added; model group: H_2_O_2_-treated group; dosing group: incomplete medium with IRP final concentrations of 100, 200, and 400 μg/mL.

Cells were divided into control, dosing, and model groups. Cells in the dosing group were replaced with different concentrations (100, 200, and 400 μg/mL) of IRP medium; cells in the control and model groups were replaced with equal amounts of medium and were incubated in the cell incubator for 24 h. The supernatant was discarded, and the cells were carefully washed with sterile PBS. The dosed and model groups were replaced with 400 μM H_2_O_2_ medium and the control group was replaced with an equal volume of medium and continued to be incubated for 4 h. Cell viability was determined using the CCK-8 method.

#### 3.6.5. Determination of ROS Generation

Cells in an optimal growth state and the logarithmic growth phase were inoculated in 6-well plates and incubated overnight. At that time, the medium was discarded, and DCFH-DA, which had been diluted with serum-free medium, was added and incubated in a 37 °C, 5% CO_2_ cell incubator for 20 min. After incubation, the supernatant was discarded, and the cells were washed three times with a serum-free medium to remove any DCFH-DA that had not entered the cells. Then, the intracellular ROS content was determined by measuring the fluorescence intensity of the cells using flow cytometry and was observed under a fluorescent inverted microscope (Olympus, Tokyo, Japan).

#### 3.6.6. DAPI Staining for Morphological Changes in Cell Nuclei

The alteration of cell morphology can have a visual concept of apoptosis. Because the cell membranes of dead and living cells have contrasting permeability to DAPI, extremely low concentrations (0.5 ug/mL) of DAPI can bind to the DNA of dead cells and produce bright and stable fluorescence. DAPI is nontoxic to living cells, does not alter the ultrastructure of organelles, and has a long fluorescence retention time [32]. After obtaining the cultured cells, we removed the supernatant, washed the cells once with PBS buffer solution, and then added 4% tissue cell fixative and fixed for 30 min at 4 °C. On finishing the fixation, the cells were washed three times with PBS buffer solution. DAPI solution was added and stained at 26 °C for 10 min. The supernatant was aspirated, and the cells were washed three times with PBS buffer solution in the dark to remove any DAPI solution that had not gotten into the cells. The cells were observed under a fluorescent inverted microscope.

#### 3.6.7. Detection of Apoptosis by Flow Cytometry

The ANNEXIN V-FITC/PI Apoptosis Assay Kit was used for the detection of apoptosis. The treated cells were collected, mixed with 5 µL of Annexin V/FITC, and incubated for 5 min at 26 °C and protected from light; subsequently, 5 µL of propidium iodide solution (PI) was added, and 400 µL of PBS was used for the analysis by flow cytometry.

### 3.7. Statistical Analysis

Data are shown as mean ± SEM, and statistical analysis was performed using Origin 2021, Graphpad Prism 8, and SPSS 22.0 software. To compare the differences between the multiple groups, a one−way analysis of variance (ANOVA) was performed in this software. *p* < 0.05 indicates statistical significance.

## 4. Discussion

Antioxidants are increasingly being used as functional food components and nutritional supplements. Oxidative reactions can damage proteins, lipids, and DNA due to an uneven ratio of antioxidants to free radicals. The capacity of antioxidants to stop oxidative deterioration in food and pharmaceutical items, as well as in the body and against disease processes brought on by oxidative stress, has heightened interest in them. Appropriate techniques that concentrate on the kinetics of the reactions involving the antioxidants and address the mechanism of antioxidant activity are needed for screening the antioxidant capabilities of plants and compounds made from plants. Numerous examinations into the connection between food and human health were carried out using diverse study focus samples [9,33,34].

In many biological processes, polysaccharides act as “biological response modifiers” and are big molecules with intricate chains of aldoses or ketoses joined by glycosidic linkage. Fungal polysaccharides are used as antioxidants, antitumor agents, antiviral agents, anticoagulants, and immunostimulants. Besides this, there is mounting evidence that the majority of polysaccharides have strong antioxidant properties. When compared to ordinary small molecule molecules, polysaccharides have broad polarity, complicated structures, and large molecular weights, and their biological activity spans a wide range of domains [35,36,37]. According to research [38], polysaccharides can effectively scavenge free radicals by regulating and boosting the activity of antioxidant enzymes. The cell model is a newly developed method for studying antioxidants that can be used to quantify the antioxidant capacity of bioactive compounds in cell cultures. A mature erythrocyte is a highly differentiated cell that lacks a nucleus, organelles, and metabolic pathways. Its surface is rich in unsaturated fatty acids that are easily peroxidized [39]. At 37 °C, AAPH decomposes to generate alkyl radicals, which are converted to alkyl peroxyl radicals or alkyl peroxides, which attack cell membrane components and cause hemolysis [28,39,40]. The induced lipid peroxidation of peroxyl radicals causes erythrocyte membrane integrity loss under oxidative stress.

**Figure 16:**
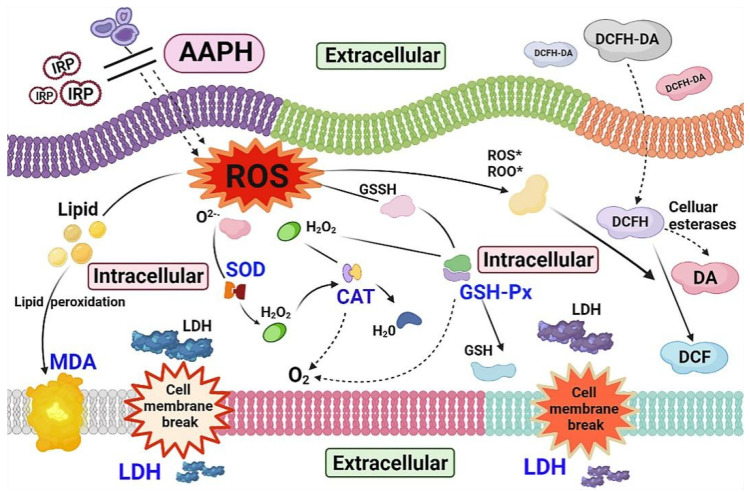
Possible intracellular antioxidant mechanisms of IRP in an AAPH-induced model of oxidative damage (the figure was produced with the online software called BioRender).

In response to the external stimuli of oxidative stress, organisms protect themselves from damage by activating a complex system of enzymes (CAT, SOD, and GSH-Px) to scavenge excess ROS [41,42]. The body’s primary antioxidant enzyme, SOD, catalyzes the conversion of superoxide anions (O_2_^−^·) into hydrogen peroxide (H_2_O_2_), whereas CAT and GSH-Px catalyze the conversion of H_2_O_2_ into water and oxygen, preventing harm to the body [43,44]. SOD, CAT, and GSH-Px rapidly eliminate oxygen radicals by interacting with each other and blocking the chain reaction of the free radicals. LDH is a cytoplasmic enzyme found in blood cells and major organ systems. This cytoplasmic enzyme is rapidly released into the cell culture medium if the cell is lysed or the cell membrane is damaged, thus serving as a suitable marker for cell damage and cell death. MDA is a natural product of lipid oxidation in living organisms. Lipid oxidation occurs when oxidative stress occurs in animal or plant cells [45,46]. Figure 16 depicts the potential intracellular antioxidant processes by which IRP reduces the oxidative damage brought on by AAPH. A free radical chain reaction is started when erythrocytes are treated with AAPH, and a lot of ROS are produced. Cellular antioxidant enzymes (CAT, GSH-Px, SOD) can constitute an intracellular defense system that coordinates the elimination of ROS, such as superoxide anions and peroxides, through a series of chain reactions [47]. Excessive cellular ROS can induce oxidative stress, which can result in lipid peroxidation, protein aberrations, and DNA breaks, which can lead to erythrocyte apoptosis [19]. In the present study, the presence of IRP effectively blocked ROS production, inhibited MDA formation, reduced LDH leakage, and modulated CAT, GSH-Px, and SOD activities, as well as IRP, which increased antioxidant enzyme activity, gradually approaching that of normal erythrocytes. The findings could be attributed to ability of IRP to prevent AAPH entry into erythrocytes, thereby reducing the need for high activity. Preincubation with IRP is, thus, effective in preventing severe changes in AAPH-induced oxidative stress enzymes and nonenzymatic defense systems, as well as in repairing AAPH-induced oxidative damage to erythrocytes. 

There is now an increasing number of researchers establishing human ex vivo models to find the most suitable model for predicting the in vivo response; among them, the immortalized cell line, HepG2, and the H_2_O_2_-induced oxidative stress model for HepG2 cells, which have been widely used to investigate the effects of various compounds on cytotoxicity [19,48,49].

HepG2 cells are highly developed, nonvirally-infected cells with metabolic and biotransformation functions resembling those of primary human hepatocytes [50]. Free radical production increases in the presence of oxidative damage inducers, but this damage can potentially be repaired in the presence of suitable antioxidants [9,51]. H_2_O_2_ can cross cell membranes easily via water channel proteins or simple diffusion and cause lipid peroxidation as well as DNA and protein damage, leading to significant oxidative damage [48,52]. Apoptosis is a form of programmed cell death that plays an important role in normal cell cycle turnover, embryonic development, immune system functioning, and cell death. Apoptosis is usually characterized by DNA fragmentation, chromatin condensation, cell shrinkage, the appearance of foaming, and the formation of apoptotic vesicles, but the cell membrane remains intact [18,52]. In addition, excess ROS causes the oxidation of proteins and lipids, which, in turn, leads to the disruption of nuclear DNA and mitochondrial integrity and, ultimately, cell death [53,54]. IRP can counteract these negative effects by reducing the level of intracellular ROS. It is noted from the experiment that 400 μM H_2_O_2_ reduced the survival rate of the HepG2 cells. This study confirmed that cells exposed to H_2_O_2_ produced large amounts of ROS, nuclear crinkling, increased early apoptosis, and late apoptosis rates (in the HepG2 cells); however, when the HepG2 cells were pretreated with IRP, H_2_O_2_-induced intracellular ROS accumulation was significantly attenuated, the number of intracellularly formed apoptotic vesicles was reduced, and the apoptosis rate decreased. Kuang reported that polysaccharides from *Agaricus bitorquis* could attenuate H_2_O_2_-induced oxidative stress in HepG2 cells by removing excess ROS and inhibiting the activities of SOD, CAT, and GSH based on the cell model method [55]. Interestingly, Tianshan green tea polysaccharides resulted in Keap-1/Nrf2 dissociation and subsequently increased HO-1 expression, causing upregulation of downstream antioxidant enzymes, such as SOD and CAT, which neutralizes ROS and has a protective effect against oxidative stress [56]. Many examples demonstrated that the *sanghuang* polysaccharides, which are capable of balancing the overproduction of ROS and protecting living organisms against these diseases through capturing free radicals and/or promoting antioxidant enzymes activities, have been pursued as potential ROS scavengers and antioxidants [36]. Further research is required to confirm that the mechanism of IRP oxidative damage protection is not only related to ROS scavenging capabilities but also to other regulatory pathways. 

Our study demonstrates, for the first time, that IRP scavenges free radicals and effectively ameliorates AAPH-induced erythrocyte hemolysis, and H_2_O_2_-induced oxidative damage in HepG2 cells by attenuating the production of oxidative stress. Hence, the results of this study provide a scientific basis for the antioxidant effect of IRP. This interesting study suggests that IRP may be a good candidate as a preventive antioxidant and hopefully, in the future, it may be used as a natural antioxidant for the treatment of various diseases associated with oxidative stress.

## 5. Conclusions

Polysaccharides with a purity of 85.40 ± 1.58% were isolated and purified from *Ischnoderma resinosum,* firstly through an ultrasonic microwave coextraction method, consisting mainly of mannose, glucose, galactose, and L-fucose, which has a triple helix structure. IRP possesses some scavenging activity against hydroxyl radicals, superoxide anions, ABTS, and DPPH radicals. In this regard, IRP manifests superior intracellular antioxidant activity in erythrocytes, protecting cells from oxidative damage by raising the activity of intracellular enzymatic antioxidant defense systems and the level of nonenzymatic antioxidant systems, thereby alleviating AAPH-induced hemolysis. IRP restrained the synthesis of ROS and MDA in combination with diminishing LDH leakage, which maintained the normal activity of cellular antioxidant enzymes (SOD, CAT, and GSH-Px) (*p* < 0.05). The IRP was able to significantly alleviate the H_2_O_2_-induced decrease in HepG2 cell survival by dampening their nuclear morphology, inhibiting ROS production, and minimizing apoptosis to safeguard the cells from oxidative damage (*p* < 0.05). To the best of our knowledge, this is the first study of the intracellular antioxidant activity of the polysaccharides of *Ischnoderma resinosum*. The preceding studies, for the first time, show that IRPs have more powerful antioxidant activity in both of the constructed models of cellular oxidative damage. This report provides a valuable scientific basis for the use of IRP as a native antioxidant.

## Data Availability

Not applicable.

## Sample Availability

Samples of the compounds are not available from the authors.

## References

[1] Zhang Y., Wang D., Chen Y., Liu T., Zhang S., Fan H., Liu H., Li Y. (2021). Healthy function and high valued utilization of edible fungi. Food Sci. Hum. Wellness.

[2] Zmitrovich I., Arefyev S., Bondartseva M., Belova N., Khimich Y., Isaeva L., Kapitonov V., Vlasenko V., Volobuev S., Ezhov O. (2019). Profiles of Little-Known Medicinal Polypores: Haploporus odorus (Agaricomycetes). Int. J. Med. Mushrooms.

[3] Mendoza L. (2002). Dictionary of the Fungi, ninth edition, by P.M. Kirk, P.F. Cannon, J.C. David, and J.A. Stalpers, 2001, CAB International. Mycopathologia.

[4] M A Fitra Z.T.S.E. (2019). The potency of mushrooms as food alternative in the forest park of Pocut Meurah Intan, Saree, Aceh Besar. Electrochem. Soc..

[5] Mittler R. (2017). ROS Are Good. Trends Plant Sci..

[6] Liu Z., Ren Z., Zhang J., Chuang C., Kandaswamy E., Zhou T., Zuo L. (2018). Role of ROS and Nutritional Antioxidants in Human Diseases. Front. Physiol..

[7] Xu X., Li S., Zhang R., Le W. (2022). Neuroprotective effects of naturally sourced bioactive polysaccharides: An update. Neural Regen. Res..

[8] Furger C. (2021). Live Cell Assays for the Assessment of Antioxidant Activities of Plant Extracts. Antioxidants.

[9] Gulcin İ. (2020). Antioxidants and antioxidant methods: An updated overview. Arch. Toxicol..

[10] Wang Z.J., Xie J.H., Nie S.P., Xie M.Y. (2017). Review on cell models to evaluate the potential antioxidant activity of polysaccharides. Food Funct..

[11] Ye B.O., Ji Y., Yuan Q., Zhang G.R., Fan Q., Wei G., Yin Z., Tao L. (2016). Sevoflurane inhibits the antioxidant capacity of erythrocytes. Exp. Ther. Med..

[12] Xu J., Zhang Y., Ren G., Yang R., Chen J., Xiang X., Qin H., Chen J. (2020). Inhibitory Effect of Delphinidin on Oxidative Stress Induced by H2O2 in HepG2 Cells. Oxid. Med. Cell. Longev..

[13] Huang S., Huang G. (2021). Extraction, structural analysis, and activities of rice bran polysaccharide. Chem. Biol. Drug Des..

[14] Zhao T., Mao G., Feng W., Mao R., Gu X., Li T., Li Q., Bao Y., Yang L., Wu X. (2014). Isolation, characterization and antioxidant activity of polysaccharide from Schisandra sphenanthera. Carbohyd. Polym..

[15] Lin D., Xu C., Liu Y., Zhou Y., Xiong S., Wu H., Deng J., Yi Y., Qiao M., Xiao H. (2022). Chemical Structures and Antioxidant Activities of Polysaccharides from Carthamus tinctorius L.. Polymers.

[16] Guo X., Kang J., Xu Z., Guo Q., Zhang L., Ning H., Cui S.W. (2021). Triple-helix polysaccharides: Formation mechanisms and analytical methods. Carbohyd. Polym..

[17] Guo Y., Chen X., Gong P. (2021). Classification, structure and mechanism of antiviral polysaccharides derived from edible and medicinal fungus. Int. J. Biol. Macromol..

[18] Al-Oqail M.M., Farshori N.N., Al-Sheddi E.S., Al-Massarani S.M., Siddiqui M.A., Al-Khedhairy A.A. (2020). Petroselinum sativum protects HepG2 cells from cytotoxicity and oxidative stress induced by hydrogen peroxide. Mol. Biol. Rep..

[19] Zhao Y., Liu S., Sheng Z., Li X., Chang Y., Dai W., Chang S.K., Liu J., Yang Y. (2021). Effect of pinolenic acid on oxidative stress injury in HepG2 cells induced by H2O2. Food Sci. Nutr..

[20] Xie D., Yuan P., Wang D., Jin H., Chen H. (2017). Effects of naringin on the expression of miR-19b and cell apoptosis in human hepatocellular carcinoma. Oncol. Lett..

[21] Xu N., Sun Y.H., Guo X.L., Liu C., Mao Q., Hou J.M. (2017). Optimization of ultrasonic-microwave synergistic extraction of polysaccharides fromMorchella conica. J. Food Process. Pres..

[22] Dubois M., Gilles K.A., Hamilton J.K., Rebers P.T., Smith F. (1956). Colorimetric Method for Determination of Sugars. Anal. Chem..

[23] Liu Y., Mo X., Tang X., Li J., Hu M., Yan D., Peng W., Wu C. (2017). Extraction Optimization, Characterization, and Bioactivities of Polysaccharides from Pinelliae Rhizoma Praeparatum Cum Alumine Employing Ultrasound-Assisted Extraction. Molecules.

[24] Miao Y., Lin Q., Cao Y., He G., Qiao D., Cao Y. (2011). Extraction of water-soluble polysaccharides (WSPS) from Chinese truffle and its application in frozen yogurt. Carbohyd. Polym..

[25] Jahanbin K., Abbasian A., Ahang M. (2017). Isolation, purification and structural characterization of a new water-soluble polysaccharide from Eremurus stenophyllus (boiss. & buhse) baker roots. Carbohyd. Polym..

[26] Ouerfelli M., Bettaieb Ben Kâab L., Almajano M. (2018). Radical Scavenging and Antioxidant Activity of Anthyllis Vulneraria Leaves and Flowers. Molecules.

[27] Gong J., Huang J., Xiao G., Chen F., Lee B., Ge Q., You Y., Liu S., Zhang Y. (2016). Antioxidant Capacities of Fractions of Bamboo Shaving Extract and Their Antioxidant Components. Molecules.

[28] Wang Q., Luo J., Liu H., Brennan C.S., Liu J., Zou X. (2019). Protective effects of the flavonoid fraction obtained from pomelo fruitlets through ultrasonic-associated microwave extraction against AAPH-induced erythrocyte hemolysis. RSC Adv..

[29] Yu D., Zha Y., Zhong Z., Ruan Y., Li Z., Sun L., Hou S. (2021). Improved detection of reactive oxygen species by DCFH-DA: New insight into self-amplification of fluorescence signal by light irradiation. Sens. Actuators B Chem..

[30] Long X., Hu X., Pan C., Xiang H., Chen S., Qi B., Liu S., Yang X. (2022). Antioxidant Activity of Gracilaria lemaneiformis Polysaccharide Degradation Based on Nrf-2/Keap-1 Signaling Pathway in HepG2 Cells with Oxidative Stress Induced by H_2_O_2_. Mar. Drugs.

[31] Zhang J., Gao H., Zhu L., Yuan X., Yang X., Xu M., Yang Y. (2022). The Protective Effect of Trichosanthes kirilowii Peel Polysaccharide on the Oxidative Damaged HepG2 and HUASMC Cells. Genet Res (Camb).

[32] Li X., Bau T., Bao H. (2018). FPOA induces apoptosis in HeLa human cervical cancer cells through a caspase-mediated pathway. Oncol. Lett..

[33] Varışlı B., Darendelioğlu E., Caglayan C., Kandemir F.M., Ayna A., Genç A., Kandemir Ö. (2022). Hesperidin Attenuates Oxidative Stress, Inflammation, Apoptosis, and Cardiac Dysfunction in Sodium Fluoride-Induced Cardiotoxicity in Rats. Cardiovasc. Toxicol..

[34] Kucukler S., Benzer F., Yildirim S., Gur C., Kandemir F.M., Bengu A.S., Ayna A., Caglayan C., Dortbudak M.B. (2021). Protective Effects of Chrysin Against Oxidative Stress and Inflammation Induced by Lead Acetate in Rat Kidneys: A Biochemical and Histopathological Approach. Biol. Trace Elem. Res..

[35] Barbosa J.R., Carvalho Junior R.N.D. (2020). Occurrence and possible roles of polysaccharides in fungi and their influence on the development of new technologies. Carbohyd. Polym..

[36] Wang H., Ma J., Zhou M., Si J., Cui B. (2022). Current advances and potential trends of the polysaccharides derived from medicinal mushrooms sanghuang. Front. Microbiol..

[37] Mu S., Yang W., Huang G. (2021). Antioxidant activities and mechanisms of polysaccharides. Chem. Biol. Drug Des..

[38] Wang Q., Wang F., Xu Z., Ding Z. (2017). Bioactive Mushroom Polysaccharides: A Review on Monosaccharide Composition, Biosynthesis and Regulation. Molecules.

[39] Ximenes V.F., Lopes M.G., Petrônio M.S., Regasini L.O., Siqueira Silva D.H., Da Fonseca L.M. (2010). Inhibitory Effect of Gallic Acid and Its Esters on 2,2′-Azobis(2-amidinopropane)hydrochloride (AAPH)-Induced Hemolysis and Depletion of Intracellular Glutathione in Erythrocytes. J. Agr. Food Chem..

[40] Qin B., Yang K., Cao R. (2020). Synthesis and Antioxidative Activity of Piperine Derivatives Containing Phenolic Hydroxyl. J. Chem..

[41] Huchzermeyer B., Menghani E., Khardia P., Shilu A. (2022). Metabolic Pathway of Natural Antioxidants, Antioxidant Enzymes and ROS Providence. Antioxidants.

[42] Zheng L., Dong H., Su G., Zhao Q., Zhao M. (2016). Radical scavenging activities of Tyr-, Trp-, Cys- and Met-Gly and their protective effects against AAPH-induced oxidative damage in human erythrocytes. Food Chem..

[43] Zhang Y., Li W., Zhang X., Yan Y., Nie S., Gong D., Tang X., He M., Xie M. (2017). Ganoderma atrum polysaccharide ameliorates anoxia/reoxygenation-mediated oxidative stress and apoptosis in human umbilical vein endothelial cells. Int. J. Biol. Macromol..

[44] Han K. (2016). Relationships among alcoholic liver disease, antioxidants, and antioxidant enzymes. World J. Gastroentero..

[45] He J., Zhu J., Yin S., Yang X. (2022). Bioaccessibility and intracellular antioxidant activity of phloretin embodied by gliadin/sodium carboxymethyl cellulose nanoparticles. Food Hydrocolloid..

[46] Guang Wang Z.L.Q.Z., Hui Wu F.L. (2016). Enrichment of Caffeic Acid in Peanut Sprouts and Evaluation of Its In Vitro. Food Chem..

[47] Chen W., Ma J., Gong F., Xi H., Zhan Q., Li X., Wei F., Wu H., Lai F. (2018). Two novel polysaccharides from the torus of Saussurea laniceps protect against AAPH-induced oxidative damage in human erythrocytes. Carbohyd. Polym..

[48] Tan J., Li P., Xue H., Li Q. (2020). Cyanidin-3-glucoside prevents hydrogen peroxide (H2O2)-induced oxidative damage in HepG2 cells. Biotechnol. Lett..

[49] Chang X., Dong S., Bai W., Di Y., Gu R., Liu F., Zhao B., Wang Y., Liu X. (2021). Methylated Metabolites of Chicoric Acid Ameliorate Hydrogen Peroxide (H2O2)-Induced Oxidative Stress in HepG2 Cells. J. Agr. Food Chem..

[50] Hurrell T., Ellero A.A., Masso Z.F., Cromarty A.D. (2018). Characterization and reproducibility of HepG2 hanging drop spheroids toxicology in vitro. Toxicol. In Vitro.

[51] Hack C.T., Buck T., Bagnjuk K., Eubler K., Kunz L., Mayr D., Mayerhofer A. (2019). A Role for H2O2 and TRPM2 in the Induction of Cell Death: Studies in KGN Cells. Antioxidants.

[52] Su M., Yu T., Zhang H., Wu Y., Wang X., Li G. (2016). The Antiapoptosis Effect of Glycyrrhizate on HepG2 Cells Induced by Hydrogen Peroxide. Oxid. Med. Cell. Longev..

[53] Naka K., Muraguchi T., Hoshii T., Hirao A. (2008). Regulation of Reactive Oxygen Species and Genomic Stability in Hematopoietic Stem Cells. Antioxid. Redox Sign..

[54] Temple M.D., Perrone G.G., Dawes I.W. (2005). Complex cellular responses to reactive oxygen species. Trends Cell Biol..

[55] Kuang H., Jiao Y., Wang W., Wang F., Chen Q. (2020). Characterization and antioxidant activities of intracellular polysaccharides from Agaricus bitorquis (QueL.) Sacc. Chaidam ZJU-CDMA-12. Int. J. Biol. Macromol..

[56] Liu J., Lin J., Huang Z., Zheng Q., Lin F., Wu L. (2022). Chemical characterization ofTianshan green tea polysaccharides and its protective effects on cell oxidative injury. J. Food Biochem..

